# Case Report: Radiotherapy Plus Immunotherapy and Lenvatinib for the Treatment of Recurrent Hepatocellular Carcinoma With a Right Atrium and Inferior Vena Cava Tumor Thrombus

**DOI:** 10.3389/fonc.2022.879454

**Published:** 2022-05-12

**Authors:** Yuting Qian, Long Gong, Su Li, Kun Mao, Xianming Li, Guixiang Liao

**Affiliations:** Department of Radiation Oncology, Shenzhen People’s Hospital, The Second Clinical Medical College, Jinan University, Shenzhen, China

**Keywords:** hepatocellular carcinoma, immune checkpoint inhibitor, radiotherapy, lenvatinib, case report

## Abstract

**Background:**

The treatment of hepatocellular carcinoma (HCC) with right atrium (RA) and inferior vena cava (IVC) tumor thrombi is challenging, with the standard treatment being not well established. Immunotherapy plus antiangiogenic therapy is a potentially effective treatment for patients with advanced HCC. Here, we described the case of a patient with HCC with RA and IVC tumor thrombi who achieved a successful response from radiotherapy and targeted therapy plus immunotherapy.

**Case Summary:**

A 62-year-old women presented with severe bilateral lower extremity edema identified as recurrent HCC with RA and IVC tumor thrombi based on past medical history and computed tomography. The patient received palliative radiotherapy plus pembrolizumab and lenvatinib treatment and was relieved of disease symptoms of bilateral lower extremity edema. The HCC with RA and IVC tumor thrombi shrunk, and the progression-free survival of this patient was > seven months.

**Conclusion:**

Tumor thrombus-directed radiotherapy plus concurrent immunotherapy and targeted therapy might be a feasible and safe approach for patients with HCC with RA and IVC tumor thrombi.

## Introduction

Primary liver cancer is the sixth most commonly diagnosed cancer and the third leading cause of cancer-related deaths worldwide in 2020, with approximately 906,000 new cases and 830,000 deaths (1). Nevertheless, in China, hepatocellular carcinoma (HCC) is the fourth leading cause of cancer-related deaths (accounting 326,000 deaths), with 370,000 new cases in 2015 (2). HCC with a tumor thrombus extending into the inferior vena cava (IVC) or the right atrium (RA) is rare, accounting for 3.8% of the HCC cases (3, 4). In addition, vasculature invasion is a poor prognostic factor in patients with HCC (5). The median survival of patients with untreated macrovascular thrombi is approximately five months (6). The causes of mortality include heart failure, pulmonary embolism, Budd–Chiari syndrome, and other events (7).

Lenvatinib and sorafenib are both multi-kinase inhibitors. Sorafenib can simultaneously inhibit various intracellular and cell surface kinases, including rapidly accelerated fibrosarcoma (RAF) kinase, vascular endothelial growth factor receptor-2 (VEGFR-2), VEGFR-3, and platelet-derived growth factor receptor-beta (PDGFR-β). Lenvatinib and sorafenib have dual antitumor effects. On the one hand, they can directly inhibit tumor growth by inhibiting the RAF/MEK/ERK signaling pathway; on the other hand, they can block VEGFR and PDGFR. Both sorafenib and lenvatinib have survival benefits and are recommended for managing patients with advanced HCC (8, 9). However, the efficacy of sorafenib is limited due to developed drug resistance.

The major neuronal allotypes of RAF, BRAF, and MEK pathways play a key role in HCC evasion of tyrosine kinase inhibitor (TKI) activity (10). Immune checkpoint inhibitors (ICIs) have been approved as second-line therapy for HCC in patients who previously received sorafenib (11). Pembrolizumab has shown substantial antitumor activity and a favorable toxicity profile as a second-line treatment for HCC (12). Moreover, according to the IMbrave150 trial, atezolizumab plus bevacizumab is recommended for treating unresectable HCC (13). In a clinical trial (NCT03006926), 104 patients with unresectable HCC received lenvatinib orally daily and 200 mg pembrolizumab every three weeks (q3w), the objective response rate was 46%, with median overall survival (OS) of 22 months (14).

Radiation therapy, especially image-guided radiation therapy, can be used to treat liver cancer. For HCC, stereotactic body radiation therapy (SBRT) can achieve adequate local tumor control and survival benefits (15). SBRT plus immunotherapy showed a 100% response in five advanced HCC patients (16).

Here, we report a case of recurrent HCC with RA and IVC tumor thrombus that received radiation plus pembrolizumab and lenvatinib treatment.

## Case Description

**Figure 1:**
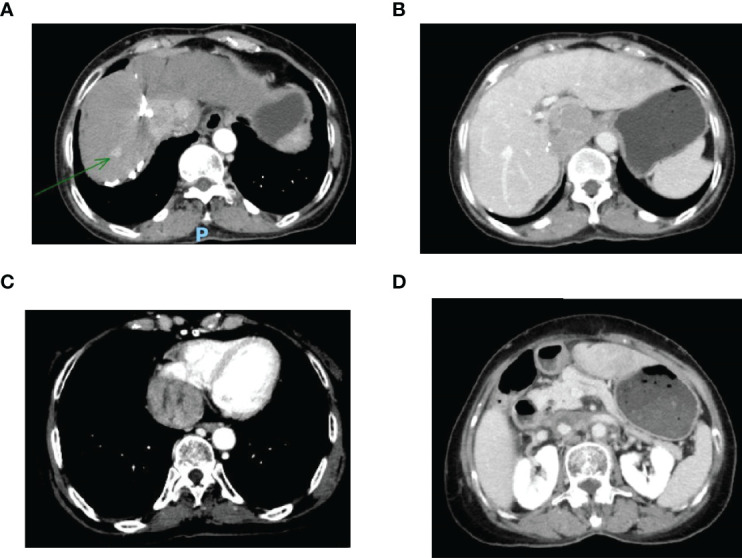
**(A)** Enhanced CT revealed a mass tumor in the S5/S8 segment of the liver; **(B)** The tumor thrombus in the inferior vena cava; **(C)** The tumor thrombus in the right atrium; **(D)** The tumor thrombus in the left renal vein. CT, computed tomography.

**Figure 2:**
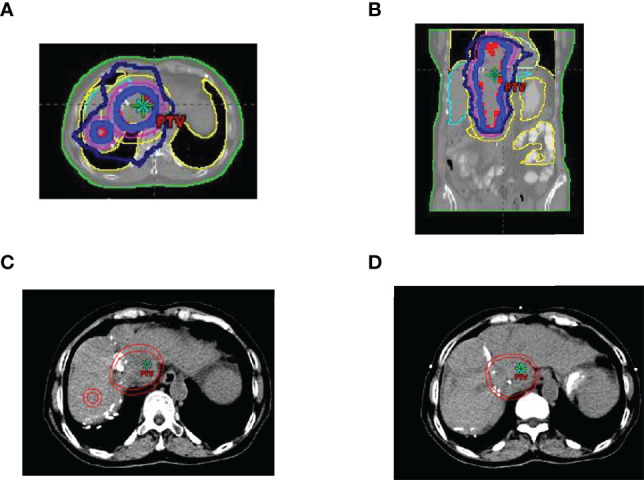
Region of image-guided radiotherapy. **(A)** cross-sectional dose distribution map; **(B)** coronal dose distribution map; **(C, D)** target delineation of gross tumor volume (GTV) and planted target volume (PTV), the inner circle line is GTV, and the outer circle line is PTV.

**Figure 3:**
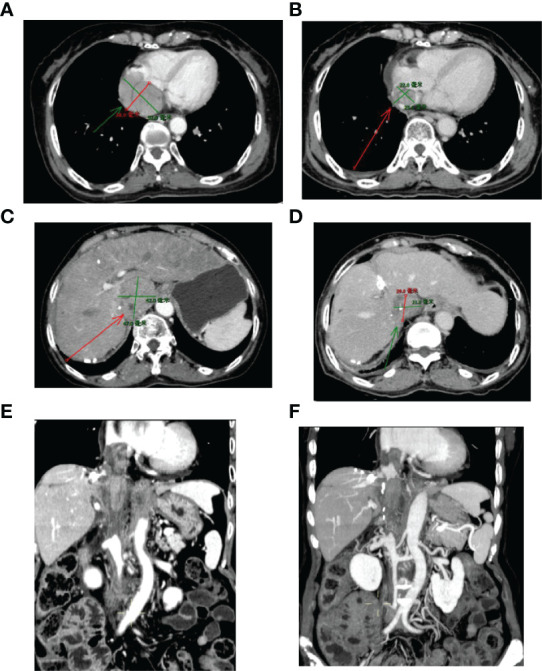
Sizes of RA and IVC tumor thrombi before and after 3-month treatment. **(A, C, E)** Before treatment; **(B, D, F)** Three months after treatment. IVC, inferior vena cava; RA, right atrium.

**Figure 4:**
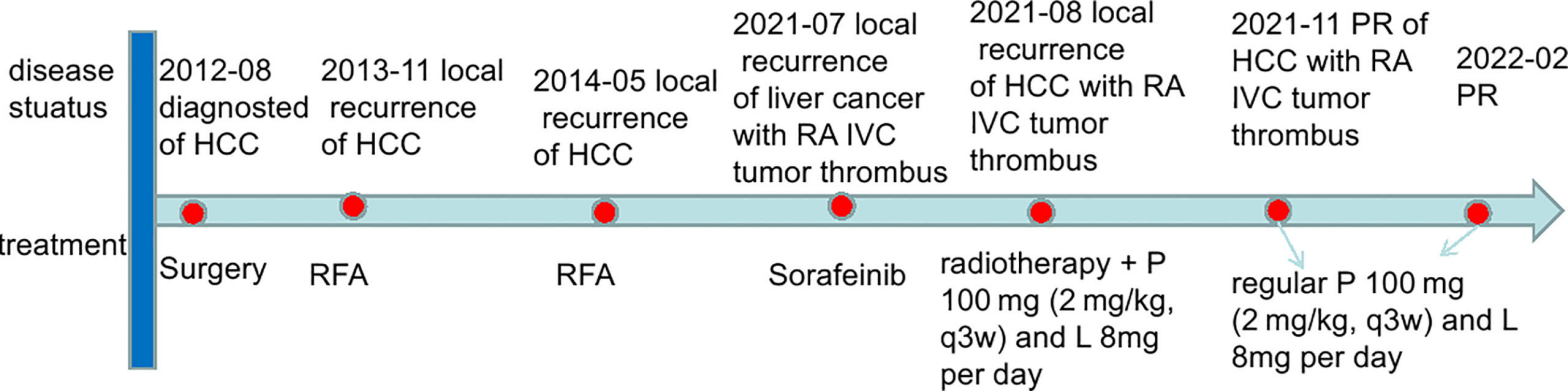
The timeline scheme of the major clinical events of the patient since diagnosis. HCC, hepatocellular carcinoma; IVC, inferior vena cava; RA, right atrium; RFA, radiofrequency ablation; P, pembrolizumab; L, lenvatinib; PR, partial response.

This case report was conducted per the CARE Guidelines (17). In July 2021, a 66-year-old Chinese woman was hospitalized due to severe bilateral lower extremity edema and palpitation, without any accompanying symptoms, such as abdominal pain or bloating. She had no history of high blood pressure, diabetes, or hepatitis. She had been diagnosed with HCC nine years ago and had undergone surgical resection. Postoperative pathology revealed a highly differentiated HCC. She had received radiofrequency ablation because of local HCC recurrence in November 2013 and May 2014. Subsequently, the patient did not undergo medical examination until July 2021. In July 2021, an enhanced computed tomography (CT) showed HCC recurrence at the junction of the anterior segment (S5/S8) of the liver, with approximately 1.8 × 1.4 cm size. The mass wrapped and invaded the adjacent IVC and grew into the IVC, RA, and left renal vein. The diagnosis was HCC with RA and IVC tumor thrombi with Barcelona clinic liver cancer (BCLC) stage-C (**Figure 1**). Then, the patient received sorafenib treatment and developed grade 4 (the US National Cancer Institute Common Terminology Criteria for Adverse Events [CTCAE v4.03]) skin rash and discontinued the sorafenib treatment. Hormones and proglobulin were used to treat dermatitis, and adverse skin reactions were reversed. According to a published study, radiotherapy can be used to treat liver cancer with RA and IVC tumor thrombi (18). Moreover, immunotherapy plus radiotherapy and antiangiogenic therapy is a safe and effective approach for advanced HCC (19). Therefore, we administrated radiotherapy, immunotherapy, and lenvatinib. The patient received radiotherapy in August 2021 through volumetric modulated arc therapy and respiratory gating technology. The dose for HCC recurrence was 50 Gy/25 fractions and for HCC with RA and IVC tumor thrombi was 45 Gy/25 fractions (**Figure 2**). She simultaneously received pembrolizumab (100 mg; 2 mg/kg, q3w) and lenvatinib (8 mg/day). Lower extremity edema and palpitations resolved after radiotherapy. Toxicity was well tolerated with no liver toxicity, and grade ≥ 3 adverse events were observed. After four cycles of pembrolizumab plus lenvatinib treatment, the CT scan indicated that the patient had a partial response and a decreased thrombus according to the Response Evaluation Criteria in Solid Tumors (RECIST) v.1.1. The HCC lesion at the junction of S5/S8 segment disappeared, and the IVC/RA thrombus decreased in size (**Figure 3**). No disease progression was observed. The patient continued to receive the pembrolizumab plus lenvatinib treatment (the last pembrolizumab treatment day was March 26, 2022). The patient remained stable at the time of writing (> 7 months). During the treatment period, there was no grade ≥ 3 adverse events or liver toxicity. Leukopenia (grade 2), thrombocytopenia (grade 1), hypoalbuminemia (grade 1), and hypertension (grade 2) were resolved using symptomatic drug treatment. Granulocyte colony-stimulating factor was administered to deal with white blood loss. A CT scan was regularly performed for every 3 months. Blood routine, liver function, kidney function, electrolyte, thyroid function, and pituitary function were regularly measured. The timeline scheme of the major clinical events of the patient since HCC diagnosis is shown in **Figure 4**.

## Discussion

In this case, the patient with HCC recurrence with RA and IVC tumor thrombi received radiotherapy, and pembrolizumab plus lenvatinib was administered to the patient. Partial responses after three months of treatment were observed. The patient was treated with (100 mg; 2 mg/kg, q3w) and lenvatinib (8 mg/day) regularly. Blood routine, liver function, kidney function, electrolyte, thyroid function, and pituitary function were regularly measured. A CT scan was regularly performed for every 3 months.

The progression-free survival (PFS) was > 7 months at the last calculation with no grade ≥ 3 adverse events.

The treatment of HCC with RA tumor thrombus *via* progression through the hepatic veins into the IVC is challenging (18). Treatment with sorafenib and best supportive care (20) as well as pembrolizumab plus lenvatinib treatment has been recommended (21). However, the prognosis of RA tumor thrombi is poor, and the identification of safe and effective local therapies is required. Local therapies include surgical resection (4), microwave ablation, transarterial chemoembolization, and radiotherapy.

A radiotherapy is an effective approach for managing unresectable HCC, with radiographic response rates of 49–98% and local control rates of 68–100% (22, 23). Bitterman et al. reported 10 patients with HCC and IVC and RA thrombi receiving a median of 50.6 Gy radiation. The median follow-up was 5 months, and no grade ≥ 3 acute toxicities occurred (18). Duan et al. reported 11 patients with IVC and RA who received transarterial chemoembolization and 60 Gy/30 fraction radiotherapy. Radiographic responses with no severe toxicity were observed in all patients. The median OS was 21 months (24). A recent review included 105 patients with HCC with IVC and RA thrombi, with a median age of 58.7 ± 12.4 years. Different groups were classified according to their treatment choices. The OS was 40.8% in these patients, indicating effective radiotherapy. The major morbidity was due to intra or extrahepatic recurrence of HCC (25). Fleckenstein et al. reported that a patient with HCC thrombus extending into the RA received CT-guided high-dose-rate brachytherapy; the patient showed relieving disease symptoms and good radiologic response (26).

Immunotherapy is a therapeutic modality in many solid tumors. Patients with advanced HCC may benefit from ICIs (21). Anti-programmed cell death-1(anti-PD-1) therapy in 413 patients with HCC was investigated in the Keynote-240 trial. The median OS was 13.9 months in the anti-PD-1 group and 10.6 months in the best supportive care group. The response rates were 18.3% and 4.4% in the anti-PD-1 and best supportive care groups, respectively. Moreover, the toxicity of ICIs was well tolerated (27). Furthermore, Keynote-224 investigated the effect of pembrolizumab in patients with advanced HCC, who had previously been treated using sorafenib, and showed that pembrolizumab might be an effective way for this group of patients (28). Sorafenib is a molecularly-targeted drug approved by the U.S. Food and Drug Administration (FDA) for treating advanced HCC. The antiangiogenic agent lenvatinib is a targeted small molecule approved for the first-line treatment of advanced HCC. However, only approximately 30% of patients with advanced HCC showed good treatment response after receiving sorafenib, and many patients gradually progressed to insensitivity or resistance to sorafenib within six months. Drug resistance involves many signaling pathways, such as the RAF, BRAF, and MEK pathways. Advanced HCC patients with BRAF mutations display multifocal or more aggressive behavior with resistance to TKI. Long non-coding RNA may play a vital role in BRAF aggressiveness in HCC (10). Tumors grow and evolve through constant crosstalk with the surrounding microenvironment. Emerging evidence indicates that angiogenesis and immunosuppression frequently occur simultaneously in response to this crosstalk (10). Accordingly, strategies combining antiangiogenic therapy and immunotherapy have the potential to balance the tumor microenvironment and improve treatment response (10). The combination of antiangiogenic therapy with immunotherapy (atezolizumab plus bevacizumab) was also demonstrated in the IMBRAVE 150 study to provide a survival benefit for advanced HCC (13). Moreover, low-dose antiangiogenic therapy and immunotherapy improved the treatment effect in patients with breast cancer (29). Two HCC patients with lung metastases treated with lenvatinib plus pembrolizumab showed a good radiologic response and median PFS of > 12 months (30). The objective response rate of 29 patients receiving ICIs plus lenvatinib was approximately 25.9%, with one patient having a complete response, 6-month OS was 62.6%, 12-month OS was 53.7%, and 6-month PFS rate was 43.5% (31).

One study included 65 patients who received lenvatinib plus immunotherapy and 45 patients who received lenvatinib monotherapy. Lenvatinib plus ICI provided significantly higher OS and PFS than lenvatinib monotherapy. The objective response rate and disease control rate were significantly higher in the lenvatinib plus ICI group than in the lenvatinib monotherapy group. No treatment-related deaths occurred. Grade 3 or higher hypertension and palmar-plantar erythrodysesthesia were observed in 20% and 10.8% of the patients in the combination group and 17.8% and 4.4% of the patients in the single-agent group, respectively (32). In another study, patients with HCC showed complete response after lenvatinib plus pembrolizumab treatment (33). An HCC patient with IVC and RA tumor thrombi and left adrenal gland metastasis received transarterial chemoembolization, immunotherapy, and subsequent radiotherapy. The patient survived for > 34 months since the disease diagnosis (34).

The tumor microenvironment has a strong immunosuppressive effect. Although there is considerable intratumoral heterogeneity in tumors, various mechanisms may play an immunosuppressive role, including altered cytokine signaling and the presence of immunosuppressive cells. Radiotherapy for tumors can transform an immunosuppressive environment into an immunostimulatory environment. Ionizing radiation can also promote both immunosuppressive and carcinogenic effects. Regulating the immune microenvironment by radiotherapy may involve the following mechanisms: (1) local production of chemokines, cytokines, and other soluble factors; (2) changes in the tumor-associated stroma and endothelium; (3) transport or regulation of immune cell subsets into the tumor microenvironment (35). Radiation activates the immune system, exerts a synergistic antitumor effect through a combination of immunotherapy, and can increase the expression of the immune cells (36, 37). Yttrium-90 radioembolization (Y-90 RE) plus immunotherapy was safe and effective in patients with advanced HCC, with an objective response rate of 31% (38). The combination of radiotherapy and immunotherapy in patients with HCC showed 1- and 2-year PFS of 93.3% and 77.8%, respectively (39). A phase I trial reported that radiotherapy plus ipilimumab had clinical benefits in 23% of patients with liver/lung cancer (40). One study included 76 patients with HCC treated with nivolumab. Of those patients, 54 (71.1%) received radiotherapy before or during immunotherapy. The patients who had received radiotherapy had significantly longer PFS and OS than patients who had not received radiotherapy (41). Some clinical trials are ongoing to evaluate the treatment of programmed cell death-1 antibody plus radiotherapy for advanced HCC. One clinical trial investigated radiation plus intravenous administration of 200 mg camrelizumab (q3w, five times since the first day of radiotherapy until disease progression or intolerable toxicity) in advanced HCC (https://clinicaltrials.gov/ct2/show/NCT04193696). The primary outcome was the objective response rate, and the secondary outcomes were OS and toxicity. Another clinical trial investigated the efficacy and safety of SBRT followed by immunotherapy compared to SBRT alone for HCC with portal vein invasion after arterially directed therapy. Patients underwent SBRT using volumetric arc therapy, and the prescribed dose was 30–54 Gy in 3–6 fractions over 1–2 weeks (https://clinicaltrials.gov/ct2/show/NCT04167293). Further research on radiotherapy plus immunotherapy for HCC is required.

In a recent randomized phase III trial, 91 patients with locally advanced cervical squamous cell carcinoma were included. Of which 43 patients received radiochemotherapy, and 48 received chemotherapy plus Endostar (an antiangiogenic agent). The study indicated that the completed response rate was significantly higher in the chemotherapy plus Endostar group than in the radiochemotherapy group (83.33% *vs.* 65.12%) (42). Whether the three treatments have synergistic effects is worth exploring. A few studies have focused on the combination of the three treatments. A study indicated that all three treatments in murine lung tumors could inhibit tumor growth and increase immune memory protecting against tumor recurrence (43).

Although radiotherapy combined with antiangiogenic therapy or immunotherapy has been extensively studied, only a few phase III controlled studies have been successfully conducted. Owing to the complex relationship between cancer cells and tumors in the tumor microenvironment, combination therapy still requires overcoming many difficulties in achieving therapeutic benefits. Further studies are necessary to determine the optimal timing and dose of treatment (44).

Regarding treatment toxicity, no evidence of liver toxicity was observed. According to the study reported by Zhong et al. (19), the combination of immunotherapy and targeted therapy often results in toxicity. Additional radiotherapy did not increase the side effects in our case. A previous study suggested that radiotherapy plus immunotherapy did not cause radiation-induced liver disease and treatment-related deaths (39).

However, this case report also has some limitations. Most importantly, the recurrent HCC, in this case, did not undergo pathological biopsy, and the PD-L1 level in the tumor tissue could not be measured.

In conclusion, radiotherapy plus immunotherapy and targeted therapy for HCC with RA and IVC tumor thrombi might be a feasible and safe approach.

## Data Availability Statement

The original contributions presented in the study are included in the article/supplementary material. Further inquiries can be directed to the corresponding author.

## Ethics Statement

The studies involving human participants were reviewed and approved by Ethic Committee of Shenzhen People’s Hospital. The patients/participants provided their written informed consent to participate in this study. Written informed consent was obtained from the individual(s) for the publication of any potentially identifiable images or data included in this article.

## Author Contributions

GL, SL, LG, and XL treated the patient. YQ, KM, and GL wrote the paper. All authors contributed to the article and approved the submitted version.

## Conflict of Interest

The authors declare that the research was conducted in the absence of any commercial or financial relationships that could be construed as a potential conflict of interest.

## Publisher’s Note

All claims expressed in this article are solely those of the authors and do not necessarily represent those of their affiliated organizations, or those of the publisher, the editors and the reviewers. Any product that may be evaluated in this article, or claim that may be made by its manufacturer, is not guaranteed or endorsed by the publisher.
